# Evaluating Differential Basic Resistive Skills Training Effects on Sprint, Jump, and Agility in Young Basketball Athletes

**DOI:** 10.3390/jfmk10030323

**Published:** 2025-08-21

**Authors:** Jorge Arede, Jack Wells, Mark Williams, Franc Garcia, Wolfgang Schöllhorn

**Affiliations:** 1 School of Education, Polytechnic Institute of Viseu, 3504-510 Viseu, Portugal; 2 Department of Sports, Exercise and Health Sciences, University of Trás-os-Montes and Alto Douro, 5001-801 Vila Real, Portugal; 3 Research Center in Sports Sciences, Health Sciences and Human Development, CIDESD, 5001-801 Vila Real, Portugal; 4 England Golf, National Golf Centre, Woodhall Spa LN10 6PU, UK; jack.wells@aru.ac.uk; 5 Department of Sport and Exercise Sciences, Anglia Ruskin University, Cambridge CB1 1PT, UK; 6 School of Sport, Rehabilitation and Exercise Sciences, University of Essex, Colchester CO4 3SQ, UK; mark.williams1@aru.ac.uk; 7 School of Psychology and Sport Science, Anglia Ruskin University, Cambridge CB1 1PT, UK; 8 Sports Performance Area, Futbol Club Barcelona, 08028 Barcelona, Spain; francgarciagarrido@gmail.com; 9 Grup de Recerca en Ciències de l’Esport INEFC Barcelona (GRCEIB), Institut Nacional d’Educació Física de Catalunya (INEFC), Universitat de Barcelona, 08007 Barcelona, Spain; 10 Institute of Sport Science, Training and Movement Science, University of Mainz, 55099 Mainz, Germany; wolfgang.schoellhorn@uni-mainz.de

**Keywords:** team sports, variation, movement variability, adolescence, bilateral asymmetry

## Abstract

**Objectives**: This study examined how differential basic resistive skills training influences basketball players’ physical performance. **Methods**: Seventeen young male athletes completed two weekly sessions of bodyweight strength exercises based on movement variability for 6 weeks. Countermovement jump (CMJ), sprinting, and change of direction (COD) tests were used to evaluate performance before and after the intervention and after a retention phase. Based on previous research, the experimental group followed a differential strength training protocol incorporating movement fluctuations to promote adaptability alongside basketball training. The control group only engaged in standard basketball training. **Results**: Bayesian analysis showed moderate improvements in sprint performance (0–20 m and 10–20 m sprinting times) for the experimental group, though these gains were not sustained during the retention phase. Slight, non-significant increases in CMJ left leg height were observed, and no significant differences were found in COD performance. **Conclusions**: The findings suggest that differential strength training may enhance sprint performance and adaptability in young athletes. However, the findings suggest that modifications to the training protocol, such as increasing training volume (e.g., from one set to three sets of 10 repetitions) or incorporating external resistance (e.g., medicine balls, kettlebells, and barbells), may further optimize performance outcomes. This study highlights the potential of differential training methods to address the multidirectional and dynamic demands of youth basketball. Future research should explore refined protocols and their long-term impact on athletic performance and retention.

## 1. Introduction

Basketball is a globally practiced team sport that necessitates the development of a wide array of technical, tactical, social, psychological, and physical skills [1]. From a physical standpoint, young basketball players typically cover between 82.5 and 87.1 m per minute during matches [2] and perform approximately 0.78 accelerations and 0.67 decelerations per minute at intensities > 2 m/s^2^ [3]. Consequently, the sport imposes demands characterized by high intensity, intermittent efforts, multidirectional movements, and unpredictable game situations.

Basketball practitioners hold the critical responsibility of designing training methodologies that enhance players’ multifaceted capabilities, enabling them to effectively meet the unique physical and cognitive demands of the sport [4]. Thus, to optimize the physical qualities of young basketball athletes, a variety of strategic training approaches have demonstrated efficacy. Among these, resistance (or strength) training has become increasingly prevalent [5,6]. This modality involves the systematic application of progressive resistive loads and training variations to enhance health, fitness, and athletic performance [7]. Despite its benefits, the implementation of such programs among youth athletes often encounters adherence barriers [8]. While external resistance tools (e.g., medicine balls, kettlebells, and barbells) are commonly used, bodyweight-only exercises represent a particularly suitable alternative for younger populations [9]. Neuromuscular training programs, frequently integrated into warm-ups before team practices, have been shown to effectively enhance athletic qualities [9]. The enhancement of physical qualities in youth athletes should be grounded in the development of fundamental resistive movement skills, with exercises such as squats and lunges serving as foundational components [9].

In recent years, innovative athletic development methodologies emphasizing variability to facilitate motor learning have emerged. Within this field, movement variability is recognized as a critical feature of skilled performance [10]. Referred to as functional variability, this concept allows for the consistent execution of skilled actions despite external perturbations or environmental changes. Relatedly, the notion of degeneracy, i.e., the ability to achieve a given task (e.g., rebounding) through various coordinative strategies (e.g., squat variations), is promoted through training methods that embrace variability [10].

A prominent example of such an approach is differential learning, which intentionally introduces variability by ensuring that no movement repetition is executed in the same way, and which excludes the use of augmented feedback [11]. In this paradigm, “errors” are perceived as beneficial, reflecting the individual characteristics of each athlete and their learning potential. Differential learning has been successfully applied across various domains of team sports, including physical, technical, and tactical development [12,13,14]. Furthermore, studies focused on muscle strength adaptations have yielded promising results in support of this method [15,16]. However, it is important to note that the majority of these investigations have been conducted in adult populations, with limited evidence available regarding its efficacy in youth athletes.

One of the few studies targeting youth populations was conducted on young female team sport athletes. Following a six-week intervention, the group exposed to varied repetitions during rotational flywheel training demonstrated greater performance improvements compared to the non-variation group [17]. Similarly, another investigation found that youth basketball players who followed a differential bodyweight-only training protocol exhibited superior gains in physical performance relative to those in a traditional training group over seven weeks [18]. While these findings are encouraging, further research is necessary to fully understand the implications of this training modality and to develop evidence-based implementation guidelines for practitioners working with youth athletes.

The typical annual training cycle for youth team sport athletes includes rest periods of two weeks or more after the competitive season, commonly during the summer months (July–August). Although such rest is vital for both physical recovery and psychosocial well-being, it often leads to partial detraining and the loss of previously acquired adaptations [19]. Investigating the effects of detraining following structured strength training programs is therefore essential for optimizing long-term athlete development. Previous studies on youth basketball players have explored the retention of physical adaptations post-training (e.g., after plyometric programs), indicating that improvements can be maintained for up to four weeks after cessation [20]. However, these investigations have primarily utilized traditional training paradigms, with little attention given to differential learning-based methods. This is noteworthy, as research indicates that while performance differences between training methodologies may be minimal during the acquisition phase, superior long-term retention is typically observed following differential learning protocols [21]. Thus, differential learning may represent a viable approach to counteract the negative effects of training interruptions among youth team sport athletes. Nonetheless, additional empirical investigation is warranted to validate this hypothesis.

The primary objective of the present study was to evaluate the effects of a differential basic resistive skills training intervention on the physical performance of young basketball players. It was hypothesized that participants in the experimental group, who completed a strength training program based on differential learning, would exhibit significantly greater improvements in physical performance than those in the control group, who followed a conventional training protocol.

## 2. Materials and Methods

### 2.1. Study Design and Participants

A convenience sample of seventeen trained male basketball players participated in the present study. The participants had a mean age of 14.2 years (±1.4), an average height of 1.66 m (±0.11), and a mean body mass of 58.1 kg (±15.7). These athletes possessed an average of 5.4 years (±2.2) of experience in basketball and were actively competing across three different age categories within the same sports club: U12 (*n* = 2), U14 (*n* = 9), and U16 (*n* = 6).

Participants were allocated either to the differential basic resistive skills training group (*n* = 9) or to the control group (*n* = 8) (Table 1). Only the under-14 athletes possessed sufficiently similar spatial and temporal capacities to maintain uniform study conditions during the intervention period; consequently, they were assigned to the differential training group. Two higher-level U14 athletes were part of the U16 team and were therefore assigned to the control group.

Throughout the intervention period, all participants adhered to a structured training schedule consisting of three 90 min basketball training sessions per week, complemented by one or two competitive matches held on weekends. To ensure the reliability and consistency of the data collected, strict inclusion criteria were applied: all players were required to be free of injury and to have completed all training sessions in the two weeks preceding the data collection phase. Furthermore, participants who missed any assessment sessions or failed to complete at least 90% of the prescribed intervention were excluded from the final analysis to preserve the integrity of the results.

Before the commencement of the study, comprehensive information about the investigation was provided to all parents, who subsequently expressed their consent by signing an agreement to participate. Approval for the study was granted by the Ethics Committee of the University of Trás-os Montes and Alto Douro, Vila Real, Portugal (approval code: 20/2019; approval date: 30 January 2019), following the principles outlined in the Declaration of Helsinki.

This study employed a non-blinded, controlled experimental design with repeated measures to evaluate the effects of a six-week differential strength training intervention on youth basketball players. To ensure participant familiarity with physical performance assessments and basic resistive skills training exercises, 20 min familiarization session was conducted one week before the baseline testing. Following the familiarization phase, the experimental group participated in a stochastic basic resistive skills training intervention, which involved the execution of various exercise modalities. Subsequently, all participants underwent a comprehensive evaluation of their physical performance, which included the measurement of countermovement jump (CMJ) height, 10 m sprint time, and agility performance. These tests were selected based on their demonstrated validity and reliability in previous research involving youth athletic populations [12]. Their practicality, portability, and feasibility for implementation in team sport environments with limited time further supported their inclusion in this study.

The intervention spanned six weeks and consisted of two weekly sessions (conducted on Mondays and Wednesdays), which were integrated into the warm-up period preceding basketball-specific training. The intervention was scheduled during the eighth and ninth weeks of the competitive season. During this time, the control group maintained their habitual basketball training and competition schedule, replicating the experimental group’s structure except for the basic resistive skills training component. Post-intervention assessments were conducted for one week following the final basic resistive skills training session. Thereafter, participants resumed their regular training routines without the inclusion of the basic resistive skills training protocol. A follow-up evaluation was performed to assess changes relative to baseline performance. Additionally, retention tests were administered one week after the conclusion of the intervention to evaluate the persistence of the training effects over time.

### 2.2. Differential Basic Resistive Skills Training

**Figure 1:**
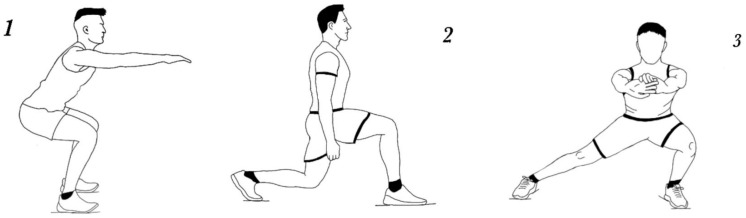
Illustration of three basic resistive skills training exercises used in the study: (**1**) bodyweight squat, (**2**) forward lunge, and (**3**) lateral squat.

Throughout 6 weeks mid-season (eighth and ninth months), athletes in the experimental group participated in two dedicated in-court training sessions per week. Each session commenced with a warm-up, followed by a series of intervention drills. These drills included dynamic stretching and neural activation exercises, which were consistently performed at the outset of each training session, post-warm-up. The core component of the training intervention, the differential strength training, involved a standardized set of own-body exercises, as visually depicted in Figure 1. Specifically, this regime entailed bodyweight squat, forward lunge, and lateral squat. The training volume consisted of 1 set of 10 repetitions (with 5 repetitions allocated to each leg) with 2 min of passive recovery following the completion of the exercise set.

The selection of tests was guided by their established feasibility in previous research involving youth athletes, particularly studies focusing on youth basketball players [18]. Before each exercise repetition, participants received standardized verbal instructions to perform a distinct movement variation, as detailed in Table 2, or a combination thereof. These variations were selected following the principles of differential learning-based training [11], ensuring that no single movement pattern was repeated within the same training session. The specific movement fluctuations were identified through a comprehensive review of prior studies that successfully implemented differential learning methodologies in motor skill development [12]. This protocol is grounded in differential learning theory, which asserts that novices should initially emphasize variability in the geometric characteristics of movement to optimize skill acquisition [11]. Importantly, the training program was implemented without any adverse events occurring throughout its duration.

### 2.3. Testing

The testing sessions for this study were conducted on a familiar indoor basketball court, identical to the facility where participants regularly trained throughout the competitive season. To ensure environmental consistency during assessments, both pre- and post-intervention evaluations commenced with a standardized 10 min warm-up protocol. Following this warm-up, participants were allowed a brief recovery period of approximately 2 to 5 min for hydration and rest before undertaking the physical performance tests. To maintain methodological rigor, all assessments were administered in a standardized sequence, consistent with established guidelines for test ordering [22]. The testing battery included bilateral and unilateral countermovement jumps (CMJs), the modified 505 agility test, and the 10 m sprint test. Throughout the data collection process, testing equipment, measurement protocols, and personnel remained constant. Three experienced sports science practitioners supervised all pre- and post-intervention sessions to minimize inter-rater variability and ensure procedural uniformity.

*Jump height.* For jump height assessment, participants performed three unilateral (single leg) and bilateral CMJs from an upright stance on an infrared contact platform (Optojump, Microgate, Bolzano, Italy), following the Bosco Protocol [23]. This protocol permitted participants to self-select the depth and speed of flexion, which were not recorded. Additionally, the CMJ asymmetry index was calculated using the formula described elsewhere [24], and the trial with the highest jump height was selected for subsequent statistical analyses.

*Change of direction performance.* Regarding the modified 505 agility test to assess change of direction (COD) performance, participants executed a sprint to a marker located 5 m from the start line, performed a 180° directional change using either the right or left leg to push off, and returned to the start line, thereby covering a total distance of 10 m [25]. Accuracy was ensured by requiring participants to fully cross the designated line with the entire foot at each turn. Completion time was recorded via photoelectric timing gates (Witty, Microgate, Bolzano, Italy) positioned at 90 cm height and spaced 1.5 m apart. Each participant completed two COD sprints per side, with a two-minute rest interval between efforts. Trials began from a standing staggered position, with the participant’s front foot placed 0.5 m behind the initial timing gate.

*Sprinting times.* Sprint performance was evaluated using single-beam photocell gates (Witty, Microgate, Bolzano, Italy) positioned 0.9 m above ground level to capture times for 10 m and 20 m distances. Participants initiated each sprint from a self-selected standing position located 50 cm behind the first photocell, which triggered the digital timer at movement onset. The protocol comprised two maximal 20 m sprints with two minutes of passive recovery between attempts. For statistical purposes, the fastest recorded times for both the 10 m and 20 m sprints were selected.

### 2.4. Statistical Analysis

Descriptive statistics, expressed as mean ± standard deviation, were computed for each variable. To evaluate the reliability of the test measures, the average measures two-way random effects intraclass correlation coefficient (ICC) with absolute agreement was calculated, accompanied by 95% confidence intervals (CIs) and the coefficient of variation (CV). ICC values were interpreted according to the classification previously proposed [26] as follows: poor (<0.5), moderate (0.5–0.74), good (0.75–0.9), and excellent (>0.9). Coefficients of variation were considered acceptable if below 10% [27].

The normality of the data distribution and sphericity were confirmed using the Shapiro–Wilk statistic and Levene’s test for the equality of variances, respectively.

Bayesian independent samples t-test (for normally distributed variables) or Mann–Whitney U tests (for non-normally distributed variables) were subsequently used to determine statistical difference between two independent groups regarding anthropometric characteristics and training experience, at the baseline (Table 1). To assess the effects of the 6-week basic resistive skills training intervention on physical performance metrics within each group, a Bayesian Repeated Measures ANOVA was conducted using a default r scale prior width of 0.5. Given baseline differences between groups, Bayesian Repeated Measures ANCOVA was employed to examine main effects of training group (experimental vs. control) and time (pre-test, post-test, retention), incorporating baseline values as a covariate. This analysis also used a default r scale prior width of 0.5.

The Bayes Factor (BF_10_) was interpreted following the evidence categories previously outlined [28]: extreme evidence supporting the null hypothesis (H0, no main effects) for BF_10_ ≤ 1/100; very strong evidence for H0 when 1/100 < BF_10_ < 1/30; strong evidence for H0 for 1/30 ≤ BF_10_ < 1/10; moderate evidence for H0 for 1/10 ≤ BF_10_ < 1/3; anecdotal evidence for H0 for 1/3 ≤ BF_10_ < 1; anecdotal evidence for the alternative hypothesis (H1) for 1 ≤ BF_10_ < 3; moderate evidence for H1 for 3 ≤ BF_10_ < 10; strong evidence for H1 for 10 ≤ BF_10_ < 30; very strong evidence for H1 for 30 ≤ BF_10_ < 100; and extreme evidence for H1 for BF_10_ ≥ 100. Only paired comparisons demonstrating at least strong evidence for H1 (BF_10_ > 10) with a percent error below 10% were considered sufficiently robust to indicate significant effects of the basic resistive skills training intervention.

All statistical analyses were conducted using JASP software, version 0.13.01 (Amsterdam, The Netherlands).

## 3. Results

All ICCs were excellent, and all the CVs were acceptable (Table 3).

**Figure 2:**
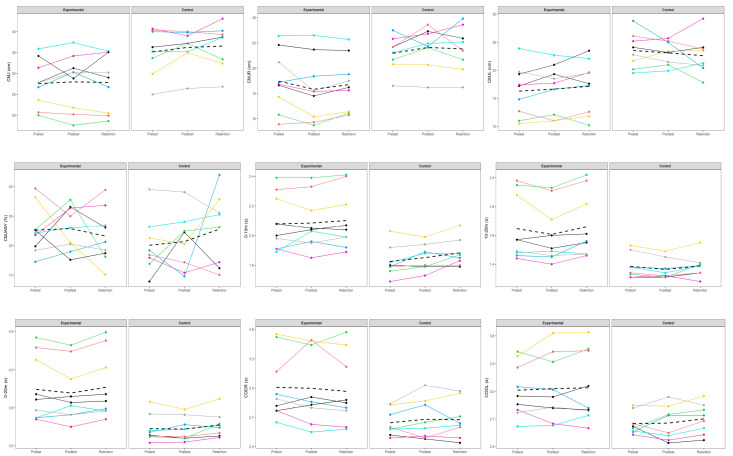
Pre- and post-intervention response comparison for each participant. Note: Each colored line represents the performance trajectory of an individual participant across the three assessment points (Pretest, Posttest, and Retention). The black dashed line indicates the group mean at each time point.

The Bayesian Mann–Whitney U test showed strong evidence for a group difference in age (BF_10_ = 10.235), while evidence for differences in experience was anecdotal in favor of the null hypothesis (BF_10_ = 0.522). For height (BF_10_ = 0.647) and weight (BF_10_ = 0.904), Bayesian independent samples t-tests also indicated anecdotal evidence for the null, suggesting no meaningful between-group differences. Figure 1 presents the individual performance changes from pre- to post-test for each training cohort. The Bayesian Repeated Measures ANOVA indicated that, within the experimental group, the null model best explained most variables, except for the 0–20 m and 10–20 m sprint times, as detailed in Table 4 and Figure 2. Specifically, the model incorporating the factor of time exhibited anecdotal to moderate evidence influencing these sprint performances, with Bayes Factors (BF_M_) of 1.14 for the 0–20 m sprint and 3.18 for the 10–20 m sprint.

Post hoc analyses for the 0–20 m sprint within the experimental group revealed anecdotal differences between pre-test and post-test (BF_10_ = 0.581) and between pre-test and retention test (BF_10_ = 0.487). However, moderate evidence (BF_10_ = 3.873) suggested a significant difference between the post-test and the retention test, indicating a potential decline in performance over time after the post-test. A comparable trend was observed for the 10–20 m sprint, with anecdotal differences between pre-test and post-test (BF_10_ = 1.38) and pre-test and retention test (BF_10_ = 0.420), alongside moderate evidence (BF_10_ = 6.948) for a difference between post-test and retention test, further suggesting a decrement in performance after the post-test.

In the control group, the null model similarly provided the optimal fit for most variables, with exceptions again noted for the 0–20 m and 10–20 m sprint times, as outlined in Table 4. The model including time yielded anecdotal to moderate evidence of an effect on these sprint times, with BF_M_ values of 3.86 for the 0–20 m sprint and 1.43 for the 10–20 m sprint, implying a more marked temporal effect, particularly for the 0–20 m sprint in the control cohort.

Post hoc comparisons in the control group demonstrated anecdotal differences between pre-test and post-test (BF_10_ = 0.972), and post-test and retention test (BF_10_ = 1.003), while moderate differences between pre-test and retention test (BF_10_ = 6.948) indicated a likely performance decline over time.

According to the Bayesian Repeated Measures ANOVA, the null model best accounted for variance in the CMJ asymmetry index (CMJASY). The optimal Bayesian Repeated Measures ANCOVA models for CMJ (bilateral and unilateral), and change of direction to the right (CODR) and left (CODL) incorporated the covariate exclusively. Notably, strong evidence supporting the alternative hypothesis (H1) was observed for the unilateral CMJ (CMJL) baseline differences between groups (BF_M_ = 11.91). For the unilateral CMJ to the right (CMJR), the best model included both group and covariate factors, whereas for the 0–10 m, 0–20 m, and 10–20 m sprint times, the models included time and covariate effects. In particular, the 0–20 m sprint time demonstrated strong evidence for H1 concerning time and covariate effects (BF_M_ = 12.39).

## 4. Discussion

This study examined the effects of differential basic resistive skills training on the physical performance of young basketball athletes. The results demonstrated that the differential basic resistive skills training intervention exerted limited influence on the majority of performance variables, with the null model providing the most appropriate explanation for the outcomes observed in the experimental cohort. Significant exceptions to this pattern were observed in the 0–20 m and 10–20 m sprint times. Specifically, Bayesian analysis of these sprint measures indicated evidence ranging from anecdotal to moderate, suggesting a temporal effect on performance characterized by a decline from the post-intervention assessment to the retention test.

Differential basic resistive skills training did not yield statistically significant improvements in jumping performance after the six-week intervention. Although a slight increase was noted in CMJ height for the left leg, these changes were not substantial enough to demonstrate a conclusive effect. In addition, the strong evidence for CMJL baseline differences between groups could have undermined post hoc comparisons. The findings are consistent with previous research [29], which showed no significant differences between four-session differential learning (DL) interventions and repetition-based protocols among physically active university students. The authors attributed the lack of effect to limited training volume (20 jumps per session across four sessions), a factor that may also explain the findings of the present study, where participants completed 15 repetitions per leg over 12 sessions. This suggests that a higher volume of training might be necessary to elicit the expected benefits of DL, especially for novice athletes. While the DL model began with technique training based on the fastest adaptation timescale (neuronal), another timescale appears to be linked to muscle metabolism. Adaptations at the neuronal level take place in the range of days, whereas in muscles this is more in the range of weeks and months [30,31,32]. Further research is needed to determine whether these timescales are influenced by other variables or longer interventions.

Contrary to these results, another study observed a 5% increase in CMJ performance among U15 football players following a DL-based intervention incorporating different kicking techniques, compared to a traditional kicking program [33]. In this case, the athletes performed a total of 144 maximal effort kicks, which may have provided sufficient stimulus for positive adaptation on the neuromuscular level. Indeed, one meta-analysis [34] found that neuromuscular training programs using bodyweight-only exercises were more effective on outcome measures in younger (<13 years) compared to older youth athletes. Where the outcomes included in the meta-analysis were related to stability-based measures (e.g., time to stabilization), the lower observed effects in the older group were considered to relate to an inadequate training stimulus for these individuals who were also taller and heavier than their younger counterparts. In contrast, however, other meta-analysis [35], which included a broader range of outcome measures, including estimates of leg power, strength, and sprint speed, found neuromuscular training programs to be more effective in older players (≥15 years) than younger (≤15 years) players of team sports. Accordingly, it may be that depending on age (and maturation status), such training induces unique responses. Moreover, concerning our results, it is therefore plausible that the performance measures did not capture the potential changes that the program did elicit, such as measures relating to stability and motor control. It is important to emphasize that all the interventions completed were related to geometry. That, in the first place, is targeted towards stability of a movement, being able to keep the variance smaller despite various disturbances. For achieving changes in speed or jumping performance the variations must provide stimuli variations in the stretch shortening process. This would need stimulation such as longer steps, higher step frequencies, running downhill or performing plyometrics (e.g., running on uneven surfaces). To achieve changes in muscle activity, the muscle would need to have a higher load. This also could be caused by sprinting with extended arms, extended legs, or abducting the arms while sprinting. In this case, the coordination provides longer levers that make higher forces necessary. Sprinting with maximum flexed elbows would be an alternative for higher movement frequencies.

In addition, it may be that the stimulus-induced by the bodyweight exercises may have been reduced by the high degree of variation that the DL method created, therefore decreasing the magnitude of the adaptive response. Accordingly, practitioners should consider factors such as the athlete’s experience level, training volume, and external loading when assessing the efficacy of DL strategies, which would correspond to the stochastic resonance principle in DL [21].

Additionally, the lack of improvement in CMJ performance was mirrored in the absence of significant changes in change of direction (COD) abilities after the differential basic resistive skills training intervention. This outcome aligns with previous findings [36], which reported no potentiation effect in vertical jumping or COD following a DL using a flywheel ergometer. Interestingly, only the repetition-based intervention in that study negatively affected performance in CMJ and COD tests, suggesting that a lack of familiarity with eccentric loading may have influenced the results. From a theoretical standpoint, DL interventions are most effective when they cover a wide range of movement patterns, thereby facilitating optimal self-organization and improved performance [37]. One of the primary challenges regarding differential learning is determining the appropriate level of variability for each individual dependent on the timescale of the dominant organism of interest. Excessive or insufficient variability can influence the training response, particularly in complex activities such as a change of direction. Nevertheless, in the present study, fluctuations were included following the participants’ level of expertise, which in this case involved beginners [11]. In other words, the fluctuations were based on movement geometry and were presumably tailored to the developmental stage of the subjects in this study [11]. Thus, a longer intervention duration may further clarify the actual effects of this approach when applied to strength training.

Regarding sprint performance, the observed changes in pre- to post-intervention times for the 0–20 m sprint may reflect neuromuscular adaptations stimulated by strength-based exercises. These adaptations could have enhanced muscle activity and neuromuscular control, which may have contributed to improved force production during basketball-specific actions such as sprinting and jumping. This adaptation translates into more explosive accelerations during fast breaks, quicker defensive recoveries, and improved capacity to create separation from opponents. However, the decline in performance observed between the post-test and retention test indicates that these gains were not retained, suggesting a lack of long-term enhancement in sprint kinematics or kinetics. This decline could be attributed to the relatively low training volume, which might have been insufficient to produce more enduring neuromuscular changes. It is also possible that the accumulated training load during the advanced stage of the season (eighth and ninth months) may contribute to residual fatigue, potentially impairing the athletes’ adaptive capacity and overall performance development.

Despite the varied responses to the DL basic resistive skills training intervention, it may be that the training elicited responses at cortical and subcortical levels of the central nervous system, which, as has been previously mentioned, were not captured in the performance testing. Both resistance training and motor skill adaptations follow similar short-term mechanisms, with increases in strength being typically associated with changes in inter-muscle coordination [38,39].

The primary limitations of this study include an insufficient training volume and intensity, which likely constrained the development of neuromuscular adaptations and restricted improvements in physical performance. Furthermore, the exclusive reliance on bodyweight exercises without the incorporation of external resistance may have diminished the training stimulus necessary to elicit meaningful adaptations. In such contexts, the inclusion of movements involving extended levers or higher velocities (e.g., plyometric push-offs or jumping lunges) is crucial to generating the requisite muscular stimuli. It is also imperative to distinguish between movement training and technical training, as each imposes distinct neuromuscular demands. Additionally, the degree of movement variability introduced was either excessive or insufficient, highlighting the inherent challenge of determining an optimal range that effectively enhances performance. Another important limitation concerns the non-random allocation of participants to groups, which may have introduced selection bias. In addition, the absence of biological maturation assessment precluded control for maturational differences that could have influenced the responsiveness to training. Finally, the lack of intensity monitoring during the intervention hindered the precise quantification of the training load to which participants were exposed. These considerations underscore the necessity for more refined and targeted training protocols in future investigations. Another potential limitation pertains to the impact of residual fatigue associated with mid-season testing. Lastly, the heterogeneity in participants’ ages, encompassing three distinct age groups (U12–U16), may have introduced confounding effects related to maturity status. Future studies should consider increasing the sample size within a single age category and conducting the intervention at the onset of the competitive season to mitigate the influence of cumulative basketball-specific load.

## 5. Conclusions

This study represents a novel contribution to the field, examining the effects of a differential basic resistive skills training program on the physical performance of young basketball athletes. Although the intervention demonstrated limited effects across most performance variables, Bayesian analysis provided anecdotal-to-moderate evidence of time-related effects in sprint performance over the 0–20 m and 10–20 m distances, while a decline in performance was observed after the retention period. Future research should examine the application of differential learning across varying temporal scales of adaptation, with careful consideration of individual variability and maturation statuses, particularly in prepubertal populations. Effective training optimization likely necessitates increased training volume and progressive external loading to facilitate neuromuscular adaptations. The findings of this study underscore the potential utility of using differential training while performing basic resistive skills in youth athletic development and highlight the need for further research to elucidate its long-term efficacy and personalized implementation.

## Figures and Tables

**Table 1:** Anthropometric characteristics and training experience of experimental and control groups. Values presented as mean ± standard deviation.

Group	Age (Years)	Height (cm)	Body Mass (kg)	Training Experience (Years)
**Experimental**	13.4 ± 1.0	162.9 ± 12.3	52.8 ± 14.4	5.1 ± 2.3
**Control**	15.2 ± 1.0	168.9 ± 9.0	64.0 ± 15.9	5.8 ± 2.3

**Table 2:** List of added movement variations performed during differential basic resistive skills training interventions.

#	Body Part	Fluctuations	#	Body Part	Fluctuations
1	Head	Head back	53	Scapula	Scapula elevated
2		Head forward	54		Right scapula elevated
3		Head back and forward	55		Left scapula elevated
4		Head rotated to the left	56		Scapula retracted
5		Head rotated to the right	57		Right scapula retracted
6		Head rotated left and right	58		Left scapula retracted
7		Head tilted to the left	59		Scapula depressed
8		Head tilted to the right	60		Right scapula depressed
9		Head tilted to the right and left	61		Left scapula depressed
10		Head circumduction to the left	62		Scapula protracted
11		Head circumduction to the right	63		Right scapula protracted
12	Eyes	Right eye closed	64		Left scapula protracted
13		Left eye closed	65	Trunk	Trunk rotation to the left
14		Blinking right eye	66		Trunk rotation to the right
15		Blinking left eye	67		Trunk rotation to the right and left
16		Blinking eyes	68		Trunk tilted laterally to the left
17		Look to the right	69		Trunk tilted laterally to the right
18		Look to the left	70		Trunk tilted laterally to the left and right
19		Look to the right + Blinking eyes	71		Trunk tilted back
20		Look to the left + Blinking eyes	72		Trunk tilted forward
21		Cover left eye with left hand	73		Trunk tilted back and forward
22		Cover left eye with right hand	74	Hands	Hands on hip
23		Cover right eye with left hand	75		Right hand on hip
24		Cover right eye with right hand	76		Left hands on hip
25		Look to the right + Cover left eye with left hand	77		Hands behind head
26		Look to the right + Cover left eye with right hand	78		Hands on forehead
27		Look to the right + Cover right eye with left hand	79		Right hand behind head
28		Look to the right + Cover right eye with right hand	80		Left hand behind head
29		Look to the left + Cover left eye with left hand	81		Hands behind back
30		Look to the left + Cover left eye with right hand	82		Right hand behind back
31		Look to the left + Cover right eye with left hand	83		Left hand behind back
32		Look to the left + Cover right eye with right hand	84		Clapping ahead
33		Simultaneous swing straight arms	85		Clap behind back
34		Alternated swing straight arms	86		Clap front and back
35	Arms	Static two arms up			
36		Two arms up with hand pronation and supination			
37		Two arms close to the torso			
38		Simultaneous forward arm rotation			
39		Simultaneous backward arm rotation			
40		Alternated forward arm rotation			
41		Alternated backward arm rotation			
42		Simultaneous arm abduction and adduction			
43		Alternated arm abduction and adduction			
44		Arms open to the side			
45		Arms open down			
46		Crossed arms			
47		Arms stretched forward			
48		Arms stretched back			
49		Right arm up + left arm down			
50		Left arm up + right arm down			
51		Left arm up + right arm to the side			
52		Right arm up + left arm to the side			

**Table 3:** Reliability data for test variables. Data are presented as values with lower- and -upper confidence limits.

Test Variables	ICC (95% CL)	CV (%) (95% CL)
CMJ (cm)	0.98 (0.97; 0.99)	3.81 (2.97; 4.65)
CMJ_R_ (cm)	0.96 (0.94; 0.98)	9.97 (7.79; 12.15)
CMJ_L_ (cm)	0.98 (0.96; 0.99)	6.10 (5.09; 7.11)
0–10 m (s)	0.96 (0.93; 0.98)	1.56 (1.15; 1.96)
10–20 m (s)	0.92 (0.86; 0.96)	4.44 (1.22; 7.67)
0–20 m (s)	0.93 (0.87; 0.96)	2.01 (0.90; 3.13)
M505_R_ (s)	0.96 (0.93; 0.98)	2.33 (1.73; 2.93)
M505_L_ (s)	0.96 (0.93; 0.98)	2.05 (1.38; 2.73)

**Table 4:** Descriptive statistics of the dependent variables and post vs. pre-intervention effects.

Variables		Pre-Test, Mean ± SD	Post-Test, Mean ± SD	Retention, Mean ± SD
CMJ (cm)	Experimental	27.57 ± 5.58	27.98 ± 6.67	27.82 ± 6.82
	Control	35.16 ± 5.55	36.23 ± 4.40	36.56 ± 5.28
CMJ_R_ (cm)	Experimental	17.42 ± 5.85	15.82 ± 6.20	16.72 ± 5.35
	Control	22.98 ± 3.38	24.11 ± 4.01	23.84 ± 4.53
CMJ_L_ (cm)	Experimental	16.28 ± 4.43	16.66 ± 4.35	17.13 ± 4.67
	Control	23.55 ± 3.13	23.11 ± 2.14	22.63 ± 3.39
CMJ_ASY_ (%)	Experimental	25.21 ± 8.05	25.79 ± 7.43	23.24 ± 9.05
	Control	22.74 ± 11.59	21.37 ± 9.63	25.56 ± 12.17
0–10 m (s)	Experimental	2.08 ± 0.19	2.09 ± 0.18	2.10 ± 0.20
	Control	1.83 ± 0.11	1.85 ± 0.09	1.88 ± 0.09
10–20 m (s)	Experimental	1.65 ± 0.22	1.61 ± 0.20	1.66 ± 0.22
	Control	1.39 ± 0.09	1.36 ± 0.07	1.39 ± 0.08
0–20 m (s)	Experimental	3.74 ± 0.42	3.70 ± 0.38	3.77 ± 0.43
	Control	3.23 ± 0.18	3.22 ± 0.16	3.28 ± 0.17
M505_R_ (s)	Experimental	3.01 ± 0.33	3.00 ± 0.37	2.97 ± 0.36
	Control	2.65 ± 0.14	2.68 ± 0.21	2.68 ± 0.19
M505_L_ (s)	Experimental	3.01 ± 0.29	3.03 ± 0.35	3.04 ± 0.38
	Control	2.65 ± 0.12	2.66 ± 0.19	2.70 ± 0.16

## Data Availability

The data that support the findings of this study are available from the corresponding author, J.A., upon reasonable request.

## Abbreviations

The following abbreviations are used in this manuscript:

ANCOVA	Analysis of Covariance
ANOVA	Analysis of Variance
BF	Bayes Factor
CI	Confidence interval
CMJ	Countermovement jump
CMJASY	CMJ asymmetry index
CMJL	Countermovement jump (left leg)
CMJR	Countermovement jump (right leg)
COD	Change of direction
CODL	Change of direction (left leg)
CODR	Change of direction (right leg)
CV	Coefficient of variation
H0	Null hypothesis
H1	Alternative hypothesis
ICC	Intraclass correlation coefficient
M505	Modified 505 Agility Test Left
